# Evaporative Cooling of Concrete Pavers Incorporating Recycled, Bio-Based and Lightweight Materials: Influence of Capillary Absorption and Density

**DOI:** 10.3390/ma19081658

**Published:** 2026-04-21

**Authors:** Amro Yaghi, Farjallah Alassaad, Stephane Ginestet, Gilles Escadeillas

**Affiliations:** 1 College of Engineering and Technology, American University of the Middle East, Egaila 54200, Kuwait; amro.yaghi@aum.edu.kw; 2 LMDC (Laboratoire Matériaux et Durabilité des Constructions), Université de Toulouse, UPS, INSA, 135, Avenue de Rangueil, 31077 Toulouse, France; sgineste@insa-toulouse.fr (S.G.); escadeil@insa-toulouse.fr (G.E.)

**Keywords:** urban heat island, evaporative cooling, concrete pavers, capillary absorption, recycled sand, hemp, lightweight aggregates

## Abstract

The urban heat island effect is strongly linked to the use of dense mineral pavements with high thermal inertia and lacking passive heat dissipation mechanisms. This article evaluates the potential of evaporatively cooled concrete pavers, based on capillary action and evaporation by incorporating recycled, bio-based, and lightweight materials to develop functional porosity. Ten paver formulations were developed using natural or recycled sand, hemp fibers and shives, and lightweight aggregates. Compressive strength, density, capillary absorption, and thermal behavior were characterized. Tests were conducted outdoors in full sunlight over 48 h in comparison with reference urban materials. The results show that capillary action alone is insufficient to induce effective cooling. The raw recycled sand formulation exhibits high capillary absorption but reaches maximum temperatures of 43–44 °C, which may be due to its low interconnected porosity that limits evaporation. Conversely, formulations incorporating bio-based materials or lightweight aggregates showed a more favorable balance between water availability, reduced density, and surface cooling performance. Hemp-based pavers reach maximum temperatures of 38–40 °C, while those incorporating expanded clay range between 37 and 39 °C, representing a reduction of 7 to 13 °C compared to bitumen and maintaining mechanical strengths suitable for pedestrian use. The results suggest that effective evaporative cooling is associated with sufficient capillary absorption, efficient water transfer toward the surface, and moderate density limiting heat storage. This study demonstrates that high capillary absorption alone does not ensure effective evaporative cooling. By systematically comparing recycled, bio-based and lightweight aggregates, the results reveal that evaporative cooling efficiency probably depends on the functional connectivity of the pore network and on a moderate material density limiting heat storage.

## 1. Introduction

The world is experiencing rapid urbanization and significant urban densification. This urbanization and densification have altered the energy balance at the Earth’s surface, causing a significant rise in local temperatures known as the urban heat island effect [1,2,3,4,5,6]. This phenomenon is characterized by a temperature difference, which can be several degrees, between urban and rural areas, particularly on summer nights. This is caused by several factors, primarily the replacement of natural and vegetated surfaces with impermeable, dark materials with high thermal inertia, such as concrete and asphalt. These materials absorb solar radiation and release the heat accumulated during the day [1,2]. This phenomenon leads to various consequences: increased energy consumption, reduced thermal comfort, and increased health risks during heat waves [3]. Beyond material performance, reducing surface temperature at pedestrian level is directly linked to outdoor thermal comfort, heat stress exposure and unequal vulnerability in dense urban areas [4]. Even with a reduction of just a few degrees in surface temperature, the thermal comfort perceived by pedestrians is significant [7].

Several strategies have been proposed to mitigate these effects. Examples include urban greening, the use of materials with high solar reflectance, and evaporative cooling. Active solutions, such as the periodic watering of surfaces, demonstrate that water evaporation temporarily reduces surface temperature. However, this method has the drawback of requiring significant water consumption and constant maintenance, which limits its large-scale application [5]. Following this, passive solutions, which utilize water stored in their porous networks for self-cooling through evaporation, are attracting increasing interest [6,8,9,10].

The principle of the cooling pavement relies on a coupling between capillary action and evaporation. The water contained in the pores migrates naturally to the surface due to capillary action and evaporates upon contact with the ambient air. This phenomenon is endothermic and consumes latent heat to lower the surface temperature of the material [11]. The efficiency of this mechanism depends directly on the porous structure of the concrete, particularly the size and connectivity of the pores. Furthermore, the hydrophilic nature of the concrete constituents can affect this coupling [12]. Therefore, to ensure sustained cooling even under dry conditions, the design of a suitable porous network is the solution, maintaining a continuous supply of water to the surface coupled with evaporation.

In parallel, and to promote the circular economy, several studies have explored the use of recycled or bio-based materials in concrete. In the context of this study, the value of these materials lies not only in their sustainability but also in certain physical properties that can influence the hygrothermal behavior of the final composite, particularly their water absorption capacity, porosity, and, in some cases, their lower density. These characteristics can modify the availability of water within the material as well as its thermal behavior under external exposure conditions. Recycled aggregates from demolition concrete can, depending on their origin and processing stage, exhibit higher porosity and water absorption capacity than natural aggregates [13,14,15,16]. These characteristics can influence the capillary behavior of the final material, not only through the inherent properties of the aggregates themselves but also through the modifications they induce in the overall microstructure of the cementitious matrix [17]. This high porosity is also applicable to lightweight aggregates. Similarly, bio-based materials such as aggregates and plant fibers modify the internal structure of the material by creating networks of interconnected pores [18,19]. However, the combined impact of these materials on the thermo-hydraulic behavior and cooling potential of paving stones through the coupling of capillary action and evaporation remains poorly documented.

The literature on permeable, water-retaining, or cooling pavements generally shows that greater water availability within the material can promote evaporative cooling, particularly when capillary absorption and water retention allow a sustained supply of water to the surface [20,21,22,23,24]. However, these studies often use global indicators, such as water absorption, retention capacity, or permeability, without systematically distinguishing between water stored in the porous matrix and water actually available for surface evaporation under solar radiation [20,21,22]. Consequently, the mechanisms explaining why some porous materials exhibit high capillarity but limited surface cooling remain only partially understood. In this context, it appears necessary to examine more closely the combined role of pore network connectivity, water migration to the surface, and the hygrothermal properties of the material in controlling surface temperature [21,22,24]. This study addresses this gap by experimentally demonstrating that capillary absorption alone is insufficient to induce evaporative cooling and by identifying the coupled role of pore connectivity, water migration and thermal inertia in controlling surface temperature.

This study aims to quantify the effects of bio-based, recycled, and porous materials on the thermo-hydraulic performance of concrete pavers. This is done to couple the capillary rise capacity with evaporation, which is reflected in the surface temperature. This study does not directly measure the connectivity of the porous network or thermophysical parameters such as thermal conductivity or specific heat; these concepts are therefore only used in the discussion as interpretive hypotheses. The objective is to identify which types of constituents appear most favorable to passive evaporative cooling at the material scale. The study concludes with a discussion of the potential benefits for urban comfort and climate equity. Several formulations were developed to study the effect of recycled sand compared to conventional sand, lightweight materials, and bio-based materials. Experimental tests allowed the measurement of density, compressive strength (as required for pavers), capillary rise, and surface temperature in real time.

## 2. Materials and Formulation

### 2.1. Selected Materials

The selection of materials was based on two criteria: porous and hydrophilic materials to promote capillary rise of water in concrete. This choice is based on literature studies showing that the capillarity and internal wettability of the porous network depend on the nature and structure of the concrete constituents [25].

The selected materials are river sand, recycled sand, hemp fibers and shives, and two lightweight aggregates—expanded clay and expanded glass. River sand was sourced from the Haute-Garonne department, France. Recycled sand was obtained from the demolition of buildings in the same region. Hemp fibers and shives were supplied by Agrofibre. Expanded clay and expanded glass were provided by national and European suppliers. These materials were selected based on their availability and their expected influence on water absorption and density. Another parameter in the material selection is their high porosity, water absorption capacity, and hydrophilic properties—key parameters for supplying water to the surface via capillary action [17,26]. River sand is the reference material, dense, with a smooth surface and low porosity. Recycled sand from crushed demolition concrete has a more angular texture, higher porosity, and greater water retention [27,28]. Bio-based materials (e.g., hemp fibers and hemp shives) were selected to improve capillary connectivity and enhance the material’s internal wettability [18,29]. Finally, lightweight aggregates (e.g., clay and expanded glass) were selected for their low density and high water absorption—key parameters for promoting capillary diffusion and the cooling capacity of porous concrete [8,30]. The main physical properties of the raw materials used in this study, as well as their origin, are given in Table 1. The other constituents used are standard in concrete, such as Portland cement CEM I 52.5 N with Metakaolin to improve durability [31]. The potential of alternative materials such as fiber-reinforced phosphogypsum composites warrants further exploration, as the literature shows that incorporating fibers can improve mechanical performance while modifying the composite’s porosity and water absorption [32,33,34].

### 2.2. Pavement Formulation

The formulations were created by combining different types of sand and additives to evaluate their influence on the physical and hydrothermal properties of the paving stones. The formulations are presented in Table 2.

The water/binder ratio was kept constant for all formulations in order to compare the different mixtures on a common basis and to limit the number of formulation variables. This choice does not mean that the porous structure was defined solely by this parameter. In porous concretes, the final porosity depends primarily on the target porosity, the particle size distribution and size of the aggregates, the volume of paste coating the grains, and the compaction conditions [35,36,37,38]. Thus, maintaining a constant water/binder ratio should be interpreted here as a methodological choice for comparing formulations and not as the optimum specific to each type of aggregate.

The samples were molded into 10 cm × 10 cm × 10 cm cubes and cured at 20 °C and 95% relative humidity for 28 days before the start of the experimental campaign.

## 3. Experimental Protocol

### 3.1. Compressive Strength

Paving stones require specific compressive strengths depending on their intended use, such as parking lots, sidewalks, pedestrian areas, etc. The compressive strength values are used here as comparative indicators of mechanical suitability. However, compressive strength alone is not sufficient to establish full compliance for actual pedestrian paving applications, which would also require additional product-specific and durability-related criteria.

**Figure 1:**
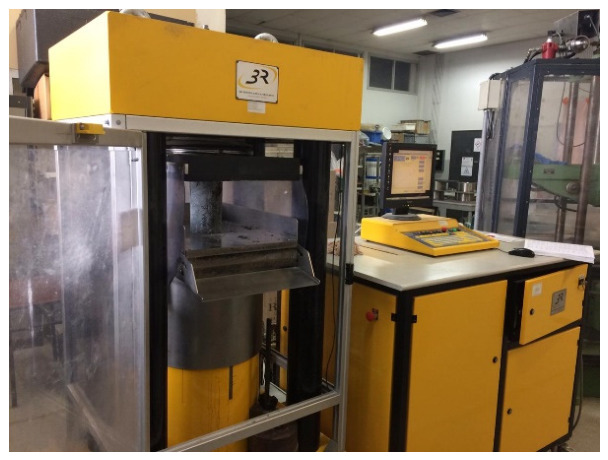
Hydraulic press “3R” used for compression testing.

To determine this, 10 cm × 10 cm × 10 cm cubes were manufactured and cured for 28 days before testing, as described in the previous section. The test is performed by applying a continuously increasing load without impact at a constant speed until the material breaks. A 3000 kN hydraulic press, “3R”, is used to apply the load to the specimens presented in Figure 1. The press is force-controlled and complies with standard EN 12390-3 [39]. The applied loading speed is set at 0.5 MPa/s. Three samples of each formulation underwent the test, and the average compressive strength was calculated by averaging the strengths obtained from three of these samples. This level of replication was selected for an exploratory comparative study between formulations; the results are therefore interpreted as comparative indicators of the mechanical behavior of the tested mixtures.

### 3.2. Density and Porosity

**Figure 2:**
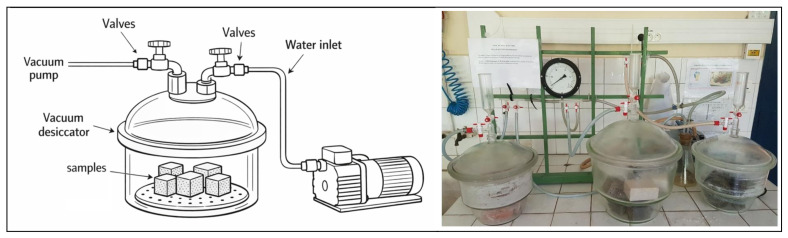
Vacuum porosity test setup.

Porosity and density were measured to study their effect on capillary rise. The tests were carried out according to standard NF P18-459 [40], as illustrated in Figure 2.

Before determining the apparent density and water-accessible porosity, the samples were saturated under vacuum in a water-filled desiccator for at least 24 h. They were then subjected to hydrostatic weighing followed by weighing in air.

The samples were dried in an oven at 105 ± 5 °C until a constant mass was reached; that is, until two successive weightings, before and after 24 h in the oven, did not differ by more than 0.05%, considered to be the dry mass. This allowed the volume of the voids initially filled with water to be obtained by subtracting the dry mass from the saturated mass. Therefore, the water-accessible porosity and apparent density are given by:

$$Density=\frac{M_{d}}{M_{a}-M_{w}}$$

$$Porosity=\frac{M_{a}-M_{d}}{M_{a}-M_{w}}$$

$M_{d}$: Dry mass (g)
$M_{a}$: Wet mass in air (g)
$M_{w}$: Wet mass in water (g)

This study does not include in-depth characterization of the pore network of the formulations, such as mercury intrusion, microscopic analysis, or direct quantification of pore distribution, size, and connectivity. Consequently, interpretations regarding the porous microstructure of the materials should be considered hypotheses consistent with the measured properties and not as direct proofs.

### 3.3. Capillary Water Absorption

**Figure 3:**
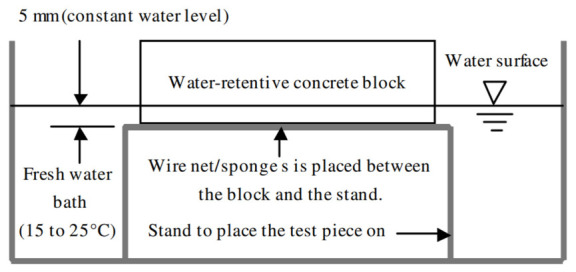
Schematic diagram of the water absorption test [11].

According to the AFPC-AFREM procedure for capillary water absorption [41], the capillary rise test consists of placing the sample on a bed of water that infiltrates the porous and hydrophilic materials by capillary action, as shown in Figure 3.

The capillary absorption coefficient is defined at each time interval x by the following relationship:

$$C_{a,x}=\frac{M_{x}-M_{0}}{A}$$

where $M_{x}$ is the mass of the specimen at a given time interval, expressed in g, $M_{0}$ is the mass of the specimen after preconditioning, and A is the cross-sectional area of the specimen (m^2^).

The origin of this rise is the pressure difference between the two sides of the pore walls. In the pores, the liquid level is higher than the level of the free surface of the container. The concave meniscus forms an angle θ with the pore surface. Capillary rise is due to the surface forces applied at every point along the meniscus contour (Laplace’s Law).

**Figure 4:**
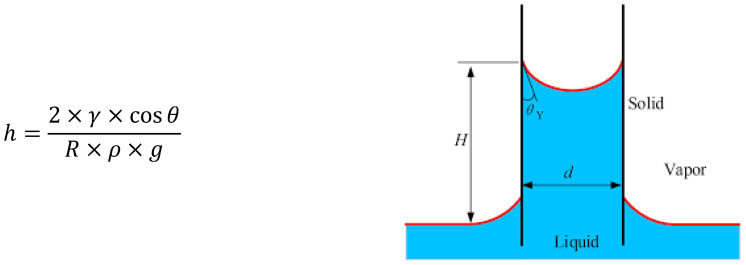
Capillary rise in a tube [42]. Where R is the inner radius of the tube (m), ρ is the density of the liquid (kg/m^3^), g is the acceleration due to gravity (9.81 m/s^2^), γ is the surface tension of the liquid (N/m), and θ is the contact angle between the liquid and the solid (degrees).

In Figure 4, Jurin’s law describes the height of this water upwelling by balancing the weight of the upwelling water and the surface tension force:

However, Jurin’s law is not entirely valid for concrete because the material also dries by evaporation. The trade-off between the capillary rise rate of water and the drying rate determines the position of the water level within the material, known as the “evaporation front.” Furthermore, the pores in concrete are influenced by their tortuosity, which can also affect water upwelling.

It should be noted that paving stones can be considered water-retaining if they meet certain conditions, such as a maximum water content in the paving stone (>0.15 g/cm^3^), with 70% of their absorption occurring within 30 min [11].

### 3.4. Real-Condition Experiment

A real-world outdoor test was conducted to evaluate the thermal behavior of different paving formulations under natural exposure. The experimental setup was installed in Toulouse, France, at the start of the summer season, around the end of May, in an unshaded area with direct exposure to sunlight. The objective of this test was to compare the surface temperature evolution of the formulations under the same exposure environment.

The samples were placed on a setup allowing water to be supplied from the underside, using a principle similar to that used for the capillary absorption test illustrated in Figure 3. Underlayment systems that could be used in real-world conditions to maintain moisture over time were not studied in this study. The test therefore aimed to compare the relative thermal behavior of the formulations under a common experimental protocol, and not to reproduce all the conditions of full-scale implementation.

In addition to the developed formulations, two reference materials were included in the test: a sample of bituminous coating and a sample of wood. These materials were selected as controls due to their contrasting thermal behaviors and were used solely as thermal comparison surfaces. No detailed physico-mechanical characterization of these control materials was performed as part of this study.

**Figure 5:**
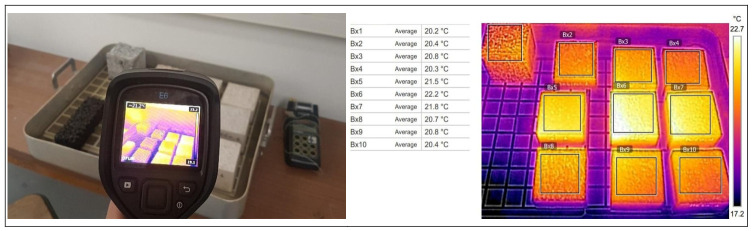
Experimental setup and a representative thermal image of the samples tested.

Surface temperature monitoring was performed using a Testo 872 infrared thermal imaging camera, as shown in Figure 5. According to the manufacturer’s specifications, this device covers a measurement range from −30 °C to 650 °C, with an accuracy of ±2 °C or ±2% of the measured value. The acquired thermal images had a resolution of 320 × 240 pixels and were recorded in a spectral band between 7.5 and 14 µm.

A thermal image of all samples was recorded hourly for 48 consecutive hours. The images obtained were then analyzed using IRSoft software, which was used with the camera. For each acquisition, a region of interest was defined on the surface of each sample, and the corresponding average temperature was extracted from the infrared image. The results presented in this study thus correspond to average surface temperatures calculated from the thermal images recorded during the test.

During the experimental campaign, the air temperature measured on-site varied approximately between 15 °C and 30 °C. However, relative humidity and solar radiation were not recorded directly on the experimental setup using instrumentation. Consequently, the test should be interpreted as a relative thermal comparison between formulations subjected to identical external conditions, and not as a complete microclimatic characterization of the test site.

## 4. Results

### 4.1. Compressive Strength

**Figure 6:**
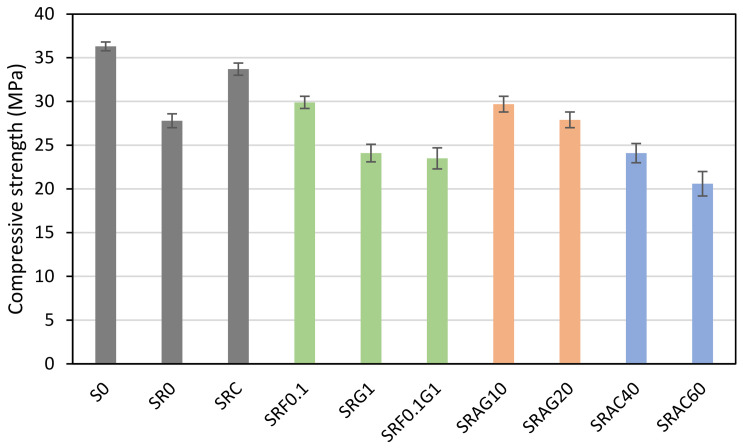
The compressive strength of the manufactured pavement.

Compressive strength is not a major concern in this project, but a minimum strength must be maintained to ensure that the paving stones are suitable for public works projects, particularly pedestrian traffic. The Japanese standard JIS A 5371 mandates a compressive strength limit of 17 MPa for concrete blocks for pedestrians [43,44]. This is why compression tests were conducted. The results are presented in Figure 6.

Firstly, although metakaolin is not the primary focus of this study, its presence in all formulations is worth mentioning. Metakaolin, considered a mineral additive, contributes to the densification of the cement paste and improves its structure. Several studies demonstrate that the pozzolanic effect of metakaolin promotes the formation of secondary C-S-H, reducing porosity and increasing durability [45,46]. In this study, despite its positive effect, it does not compensate for the decrease in strength observed with the various replacements.

The reference formulation S0, based on natural river sand, exhibits the highest compressive strength (36.3 MPa). This value is due to the good internal cohesion. However, the complete substitution of river sand with recycled sand in the SR0 formulation results in a 24% decrease in strength (27.8 MPa). This decrease is consistent with the literature, which interprets this loss as resulting from irregular morphology, increased porosity, and the presence of residual paste on the recycled sand grains, thus altering the compactness of cementitious materials [47]. Nevertheless, the use of screened recycled sand improves compressive strength compared to unscreened sand. This may seem surprising at first, but upon analysis, screening removes the finest particles and the adhering residual paste. This leads to a more homogeneous texture and better paste-aggregate adhesion [48].

Using recycled sand, the incorporation of a small amount of hemp fiber (0.1%) slightly improved compressive strength (29.9 MPa). This may be related to the bridging of microcracks and improved stress redistribution within the cementitious matrix. This phenomenon has already been discussed in studies on cementitious composites reinforced with plant fibers [49]. However, with a higher dosage and the inclusion of hemp shives, the compressive strength decreased by approximately 15% (24.1 MPa for SRG1, 23.5 MPa for SRF0.1G1). This decrease is linked to a probable increase in internal porosity and significant water absorption by the lignocellulosic constituents of hemp [50].

Regarding formulations incorporating lightweight aggregates, the trend is similar. Compressive strength decreases with the dosage of expanded aggregates, whether glass or clay. Concretes made with expanded glass (SRAG10 and SRAG20) maintain moderate strengths (29.7–27.9 MPa), while those made with expanded clay (SRAC40 and SRAC60) show a more pronounced drop (24.1–20.6 MPa). Even the lowest value remains acceptable for pedestrians because it is greater than 17 MPa [43,44]. In addition to the proportions of lightweight aggregates, this performance loss is linked to the cellular structure of these aggregates, which reduce the density and stiffness of the concrete [48,51].

### 4.2. Physical Properties: Density

**Figure 7:**
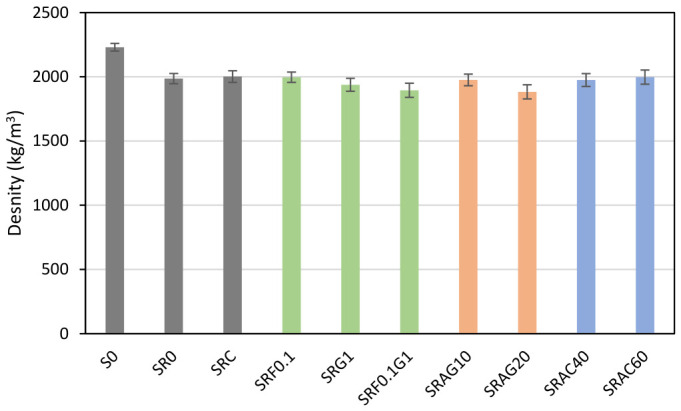
The density of the manufactured pavement.

The results presented in Figure 7 show a significant variation in density. Formulation S0 has the highest density (2230 kg/m^3^), suggesting an overall denser material than the other tested formulations, although this alone does not allow for direct conclusions regarding the fine organization of the pore network.

Replacing natural sand with recycled sand in the SR0 formulation causes a decrease in density to 1986 kg/m^3^. This is due to the increased porosity and surface roughness of the recycled grains, which trap more air and water during mixing [52]. Screening this sand slightly increased the density to 2002 kg/m^3^. This is achieved by removing fines and contaminated particles, promoting better granular compactness thanks to the homogeneous adhesion between the paste and the aggregate [53]. Formulations incorporating biomass exhibit a lower density (1895–1938 kg/m^3^). These results are consistent with the practice of substituting sand with lighter plant-based aggregates and their water absorption capacity [54]. This is also confirmed in the literature, where results show that hemp fibers improve the ductility of concrete but lead to a decrease in density proportional to the volume incorporated [49].

Finally, lightweight aggregates logically lower the density in paving stones due to their cellular structure. The difference between expanded clay and expanded glass stems from the density and strength of the aggregates themselves [51].

### 4.3. Water Absorption by Capillarity

**Figure 8:**
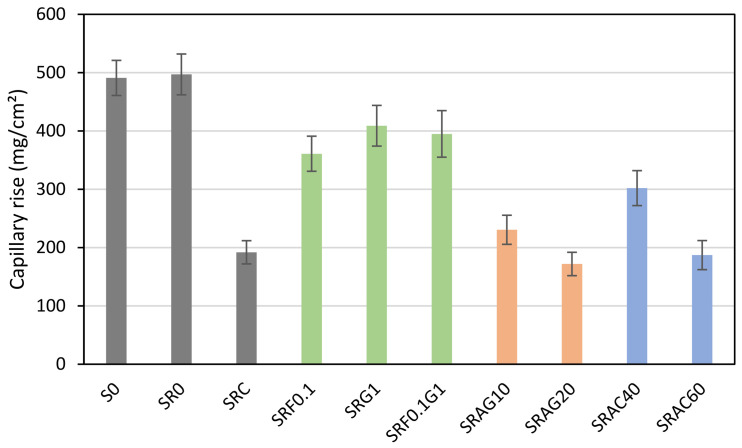
Capillary rises of different formulations.

The experimental results in Figure 8 show that capillary rise varies considerably depending on the nature of the materials used. This nature encompasses differences in open porosity, pore connectivity, and the hydrophilic or hydrophobic character of the components.

The control formulation S0, based on natural sand, exhibits a high capillary rise value (491 mg/cm^2^). This value reflects a dense microstructure with connected capillary porosity, allowing moderate water upwelling through the porous network. The introduction of raw recycled sand (SR0) leads to a slightly higher capillarity (497 mg/cm^2^), confirming the high water absorption and intergranular pore connectivity in recycled concrete. This increase is linked to the presence of residual cement paste on the recycled grains, which acts as a highly porous and hygroscopic material. Similar results were reported by Ngo et al. (2020) [52], who observed that untreated recycled materials exhibit capillary action 20 to 30% higher than that of conventional concrete.

Conversely, when the sand is screened and cleaned, the SRC formulation shows a decrease in capillary rise to 192 mg/cm^2^, representing a reduction of approximately 60% compared to SR0. According to Behera et al. (2025) [53], this improvement in capillary resistance is explained by a denser interfacial transition (ITZ) between the paste and recycled aggregates, limiting the propagation of water by capillary action.

Regarding biomass, the incorporation of hemp fibers (SRF0.1) results in a moderate decrease in capillary rise (361 mg/cm^2^ versus 497 mg/cm^2^ for SR0), representing a reduction of nearly 27%. This decrease is explained by the presence of the fibers, which reduces the size and continuity of the capillaries. However, the simultaneous introduction of fibers and hemp shives (SRF0.1G1) slightly improves the effect of the fibers: capillary rise increases to 395 mg/cm^2^. The coarser and highly porous hemp shives increase the volume of interconnected voids and the surface area for water exchange. As shown by Asghari Bareh Kheil (2024) [50] and Rahman (2024) [49], hemp exhibits high internal porosity (70–85%) and strongly hydrophilic behavior. These characteristics promote water absorption and capillary migration, but they also give the material a hygrometric regulation capacity useful for bio-based concretes subjected to wet/dry cycles.

The addition of lightweight aggregates more significantly alters the capillary behavior compared to SR0. The expanded glass-based formulations (SRAG10 and SRAG20) exhibited capillary absorption values of 230.6 mg/cm^2^ and 172 mg/cm^2^, respectively, representing a 54% to 65% reduction compared to SR0. This marked decrease reflects the almost non-absorbent nature of expanded glass, whose glassy surface acts as a hydrophobic barrier, preventing water penetration into the matrix. These results are consistent with those of Babar et al. (2020) [51], who observed a similar reduction in capillary absorption in concretes containing expanded glass. The formulations containing expanded clay (SRAC40 and SRAC60) showed intermediate performance, with capillary absorption values of 302 mg/cm^2^ and 187.2 mg/cm^2^, respectively, representing a 39% to 62% reduction compared to SR0. This behavior is attributed to the bi-porous structure of expanded clay, composed of internal closed pores and surface open pores. The former limit water penetration, while the latter allows for some capillary action. This bi-modal pore structure may contribute to a compromise between moisture transfer, thermal insulation, and moisture resistance, as demonstrated by Behera et al. (2025) [53].

### 4.4. Real-Condition Results

**Figure 9:**
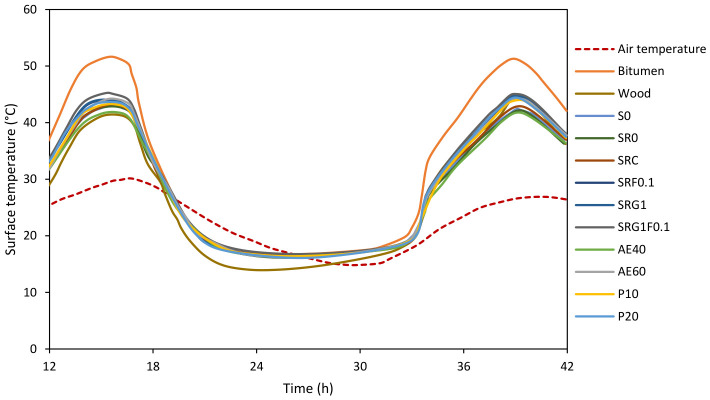
Surface temperature of different pavers exposed to real conditions.

Figure 9 shows the evolution of surface temperatures for the different formulations during a diurnal thermal cycle. Although measurements were recorded over 48 h, the first hours were excluded because the specimens were still thermally adjusting from laboratory to outdoor conditions, and the final hours corresponded to late-night conditions with limited relevance for the comparative analysis of daytime thermal response. These variations in surface temperature depend jointly on the nature of the material (structure and composition), its density, and its water absorption and evaporation capacity.

In this study, the objective is to promote capillary absorption, not to improve moisture retention, but to activate evaporative cooling, i.e., the transfer of water to the surface and its evaporation, an endothermic process that contributes to the passive cooling of the material. Formulations S0 and SR0 exhibit high densities (2230 and 1986 kg/m^3^, respectively), characteristic of a compact and conductive matrix. Their thermal behavior reflects high thermal inertia but low self-cooling capacity: daytime peaks reach 42 to 44 °C, with a slowdown in nighttime cooling. This response is typical of dense concretes: heat is stored extensively in the solid matrix and then released slowly [55]. However, SR0, although more porous than S0, does not exhibit significant cooling despite its high capillarity (497 mg/cm^2^). This apparent contradiction may be related to the fact that water absorbed within the material is not necessarily efficiently transferred to the exposed surface, which limits effective evaporation. According to recent literature, the thermal conductivity of recycled concrete cannot be explained by total porosity alone; it also depends on dry density, pore size distribution, moisture content, the characteristics of the existing bonded mortar, and the interfacial transition zone, as well as, in some cases, the connectivity of the pore network when it strongly disrupts the conduction pathway in the solid phase [56,57,58,59,60,61,62]. In this context, pore connectivity should be considered a possible contributing factor, and not the sole controlling parameter.

The SRC formulation, more homogeneous thanks to the screening of recycled sand, shows slightly more stable thermal behavior. Its moderate density (2002 kg/m^3^) and reduced capillarity (192 mg/cm^2^) reflect better internal cohesion but less free water availability at the surface. Thus, the evaporative cooling effect is less pronounced, although heat diffusion is more homogeneous.

Regarding the addition of biomass, hemp-containing formulations, particularly SRF0.1, SRG1, and SRG1F0.1, exhibit lower maximum temperatures (≈38–41 °C). Their reduced density (1895–1938 kg/m^3^) and medium to high capillarity (395–409 mg/cm^2^) promote a balance between moisture retention and gradual evaporation. Thanks to their porous and hydrophilic cell structure, hemp fibers and shives enhance capillary water migration and its release at the surface, creating an endothermic cooling effect during evaporation [63]. This phenomenon is well documented in the literature on bio-based composites: hemp acts as a natural hygrothermal regulator, absorbing moisture at night and releasing it as vapor during the day [49,50]. Thus, the decrease in surface temperatures observed for SRF0.1 and SRG1 reflects the effectiveness of the coupling between absorption and evaporation. The material remains moist on the surface for longer, which maintains a lower temperature compared to compact concretes where water evaporates rapidly or remains trapped deep within the material.

The formulations based on lightweight aggregates (AE40, AE60, P10, P20) exhibit stable thermal behavior and reduced temperature fluctuations. Their lower density (≈1880–1975 kg/m^3^) and complex porosity result in low thermal conductivity. Expanded clay (AE40, AE60), in particular, combines internal closed pores (insulating air storage) and surface open pores (capillary water migration), allowing for slow but prolonged cooling. As a result, maximum temperatures are lower (~37–39 °C), and day/night temperature variations are reduced. This synergy between thermal inertia and regulated evaporation corresponds to the optimal behavior sought for a passive cooling material [56]. Conversely, expanded glass formulations (P10, P20), whose pores are closed and non-absorbent, exhibit higher temperatures (~40–42 °C) despite their low density. This contrast confirms that porosity alone is not enough: it is connectivity and functional capillarity that determine the efficiency of evaporative cooling.

For standard or reference materials, wood stands out due to its moderate and stable temperature (~35–37 °C). Its low conductivity and natural hygroscopicity allow for balanced water absorption and release, making it a model of passive thermal regulation [64]. Conversely, bitumen reaches the highest temperatures (>50 °C), a consequence of its high solar absorptivity, dark color, and complete lack of porosity or internal moisture. These results confirm that non-porous and non-absorbent materials amplify overheating phenomena, while those with a porous and hydrophilic microstructure promote self-cooling. The microstructural mechanisms discussed in this section are offered as an interpretation of the observed trends, as the internal porous structure was not directly characterized in this study.

Finally, data on density, capillary absorption, and temperature, presented in Table 3, show a direct correlation between evaporative capacity and the reduction in surface temperature. These results clearly demonstrate that high capillary absorption alone is not sufficient to induce evaporative cooling when pore connectivity limits water transport to the surface.

These results demonstrate that effective evaporative cooling relies on three simultaneous conditions:

1. Sufficient capillarity to supply the surface with water.
2. Connected porosity that promotes outward migration.
3. Moderate density that ensures a balance between thermal inertia and permeability.

Formulations based on hemp and expanded clay meet these criteria: they offer active capillarity and suitable thermal inertia, guaranteeing gradual and sustained cooling during hot cycles.

## 5. Social and Urban Implications

The social relevance of the proposed paving solutions is directly derived from their experimentally measured ability to reduce ground surface temperatures under real outdoor conditions.

### 5.1. Thermal Comfort and the Use of Public Spaces

Thermal comfort in public spaces is an essential aspect for urban populations, especially within the broader context of the escalating frequency and severity of heat waves associated with anthropogenic climate change [65,66]. Nevertheless, indoor areas have a more comfortable atmosphere as they are more regulated by active systems, whereas outdoor thermal comfort is largely shaped by the thermo-physical characteristics of urban surface materials and their interaction with the surrounding local microclimatic conditions [67,68]. Key determinants include surface temperature, ground-emitted longwave radiation, and the capacity of urban materials to absorb, retain, and release heat [1,3].

A substantial body of research indicates that conventional and traditional mineral pavements, specifically asphalt and dense concrete, significantly contribute to pedestrian thermal discomfort. This phenomenon is attributed to their elevated solar absorptivity and notable thermal properties [65,66,69,70]. As a result, the surfaces accumulate heat throughout the day and release it gradually, resulting in elevated temperatures that are sustained until the evening and nighttime [1,55]. Therefore, the sustained heat release increases the mean radiant temperature, and the continuous heat emission experienced by pedestrians is considered a critical driver of heat stress in urban environments [65,66].

In this context, the results of this study highlight the ability of evaporatively cooled paving systems to improve thermal comfort among pedestrians in public areas. Field tests conducted in real-world conditions showed that several formulations that use recycled materials, bio-based components, or lightweight aggregates can lower the maximum surface temperature significantly compared to regular traditional paving materials. Whereas asphalt routinely exceeds 50 °C, pavers containing hemp fibers and aggregates or expanded clay maintain surface temperatures between 37 and 40 °C, corresponding to a reduction of approximately 10–13 °C depending on the formulation of the paving materials [65,66].

It is worth mentioning that even a smaller temperature reduction of approximately 3 to 5 °C, as observed in several intermediate formulations in this study (SRF0.1, SRG1, SRF0.1G1), can produce a meaningful improvement in outdoor thermal comfort. Several studies have shown that human thermal perception of outdoor areas is highly sensitive to both surface temperature and the longwave radiation emitted by ground materials [71,72]. Notably, Nikolopoulou and Lykoudis (2006) demonstrated that even a modest decrease in radiant temperature can substantially enhance perceived human comfort, particularly for individuals who are exposed for long periods in outdoor spaces, such as pedestrians, seated users, and children [73].

The thermal behaviour documented in this study derives specifically from the relationship between functional capillarity and evaporative characteristics, which together enable effective loss of heat through latent heat consumption [71]. Mixes incorporating hygroscopic constituents and well-connected porous structures, such as those mentioned in this study—hemp-based components or expanded clay—facilitate the gradual transport of moisture and its sustained evaporation at the surface. This prolonged evaporative process controls the rate of surface heating under solar exposure, in contrast to traditional dense, low-permeability materials where moisture is either retained within the matrix or evaporates too rapidly to yield a meaningful cooling effect [8,56].

Enhancements in outdoor thermal comfort extend beyond reductions in surface temperature alone [74]. By lowering the radiative heat flux emitted from the ground, these pavers effectively decrease the thermal load imposed on users, particularly on the lower limbs, which are highly sensitive during walking or prolonged standing. Prior research has consistently demonstrated that ground surface temperature plays a direct role in shaping overall thermal comfort and heat tolerance, especially within pedestrian-oriented spaces and densely utilized urban environments [3,55].

From a public-space perspective, the findings of this study indicate that the integration of evaporatively cooled paving materials can support greater and more sustained use of outdoor environments during the summer months [75]. Evaporatively cooled paving materials can lower surface temperatures; these pavers extend the period during which plazas, sidewalks, and pedestrian corridors remain comfortable, actively used and occupied, while simultaneously enhancing the perceived quality of the urban setting [71]. This is particularly relevant in high-density populations and neighborhoods, where opportunities for passive cooling through vegetation or natural shade are often constrained by limited space or low soil permeability.

Consequently, the paving formulations developed in this study (SRF0.1, SRG1, SRF0.1G1) offer a promising path for enhancing thermal comfort within public spaces, functioning as an effective supplement to the common and established urban cooling measures such as vegetation, shading structures, or reflective materials. The advantage of the proposed approach lies in its direct action at ground level, where heat exchange between pedestrians and the surface is immediate, as well as its potential compatibility with existing urban configurations. However, unlike strategies based on shading or vegetation, its thermal efficiency depends on the availability of water near the surface, which may limit its feasibility in certain contexts. This approach should therefore be considered a complementary solution, particularly relevant in sites where planting trees or installing shading devices is constrained by existing infrastructure or a lack of space [76,77,78].

### 5.2. Public Health and Urban Vulnerability—Urban Vulnerability and Its Public Health Consequences

Urban heat islands increase the ambient air temperature around them and create a significant risk to public health [79]. Numerous studies conducted to understand how prolonged exposure to high outdoor temperatures increases rates of both morbidity and mortality have demonstrated a clear correlation between high outdoor temperatures and increases in morbidity and mortality rates during extended periods of high temperatures during the summer months [4,80,81]. Furthermore, building materials that are heavy, dark, and heat-absorbing can raise both the surrounding air temperature and the temperature of the ground below them in high-density areas with many buildings [70,81].

The urban surface is one of the key factors in this process. Traditional asphalt and concrete materials can reach temperatures of more than 50–60 °C in summer, as shown in the experimental results of this study, where bitumen performed better than other materials. At these temperatures, pavements emit substantial longwave radiation and promote convective heat transfer, collectively increasing the thermal burden experienced by people occupying outdoor public spaces [80]. Such high temperatures are responsible for heating the air through convection and radiation, thus increasing the heat load on users of public spaces [3]. The high heat load is identified as a contributing factor to heat stress, which causes cardiovascular, respiratory, and neurological disorders, especially among individuals with pre-existing health conditions, children, and the elderly [3,81].

The vulnerable population includes the elderly, children, those with chronic diseases, and those who spend long periods of time in outdoor environments (urban workers, homeless persons), who have low thermoregulatory capacity or high exposure to extreme weather conditions [4,81,82,83], which increases their health risk during periods of high temperature exposure, especially in the summer months [3]. Therefore, decreasing urban soil temperature is an effective method of limiting exposure to heat through an indirect approach.

The outcome of this research indicated that some of the evaporatively cooled paving materials may achieve a reduction of 7 to 13 °C in maximum surface temperature when compared to asphalt pavement, as well as several degrees when compared to conventional concrete pavement [66]. However, even when the reduction is less dramatic, as in the case of some hemp-based and lightweight aggregate products where a reduction of 3 to 5 °C may be achieved, the implications for public health can be substantial, as epidemiological studies have shown that even a reduction of a few degrees in radiant temperature can lead to a reduction in heat wave mortalities [1,3].

The advantage of the analysed paving stones is their ability to act locally and as close as possible to people through a passive evaporative cooling mechanism. In contrast to active cooling methods like frequently watering the road or air conditioning the city, these paving stones do not require any external energy or human input [84]. The cooling effect is achieved through the gradual evaporation of water that migrates to the surface by capillary forces, which is an endothermic process and hence controls the increase in material temperature under the effect of solar irradiation [8,56].

This is especially appealing in disadvantaged urban areas because access to active cooling is often restricted. Previous studies have found a direct association between socio-economic vulnerability and heat exposure in cities, especially due to high building density, lack of vegetation, and large areas of solid impermeable surfaces [3]. In these areas, passive solutions like those integrated into existing urban infrastructures, such as self-cooling paving stones, could help reduce inequalities in heat exposure.

Additionally, lowering urban soil temperature may help reduce the effects of heat on health, specifically regarding how heat can degrade the quality of air [85]. Higher temperatures will help create tropospheric ozone and increase the amount of fine particulate matter suspended, which has been shown to negatively affect respiratory health [3]. Rain-cooling paving stones may indirectly lower surface temperatures and contribute to physically improving the microclimate and environmental conditions in urban areas [80,83,84].

Nevertheless, while the above materials do not provide a comprehensive solution for addressing urban heat health effects, they can be effective if considered as part of a more complex adaptation plan that includes greening, shading, water management and urban design [84]. Moreover, this research supports that the use of paving stones made from recycled, biologically based, and/or functionally porous materials within an urban environment can play a crucial complementary role in reducing localized heat exposure and improving health conditions throughout public spaces.

Therefore, the paving stone products that have been studied in this research may have actual potential in supporting the resilience of the health of urban areas in the face of extreme heat by reducing heat stress via their contribution to lower surface temperatures (i.e., the temperatures of flooring and roofs).

### 5.3. Urban Integration and Boundaries

The issue of incorporating evaporative cooling paving materials in the urban environment must be addressed with a systemic approach, taking into account not only the actual characteristics of such materials but also their implementation [20,86,87]. The results of this study have shown that there is indeed a possibility of a notable reduction of surface temperature using some of the proposed formulations made from recycled or bio-sourced and functionally porous materials [72,87]. Nevertheless, the actual efficacy of the proposed materials in urban settings relies on various factors, such as technical, environmental, and contextual elements that require further examination.

From an urban integration standpoint, the paving stones have a significant advantage, as they are consistent with existing urban planning typologies, such as sidewalks, urban squares, pedestrian areas, or urban courtyards, without the need to significantly alter urban uses or forms. Contrary to other cooling materials, which must have visible elements or significant alterations, the paving stones in this study are passive, invisible, and unobtrusive, relying on their physical properties [88]. This is an advantage in terms of their acceptability and compatibility with urban renewal schemes, even in constrained environments.

However, it is important to note that for optimal performance of such an evaporative cooling mechanism, water should be present within the porous structure of the material [89]. For this particular study, real-world experiments were carried out without the implementation of sub-base systems that can provide a source of continuous moisture, thereby evaluating the intrinsic effects of the paving formulations. For real-world applications, a sustained evaporative cooling effect would require a coupled hydraulic and structural design capable of temporarily storing water and passively replenishing the surface by capillary action, while preserving the mechanical function of the base and foundation layers [1,2,3,4,5]. Without periodic recharge from rainfall, subsurface moisture, or controlled water input, such cooling cannot be maintained over time. This aspect should therefore be considered a design requirement, not a result demonstrated by this study [20,24,88,90].

Yet another limitation concerns long-term performance durability. The capillary action and the functional porosity may be affected by clogging, wet/dry cycles, pedestrian traffic, or urban soiling. Although promising results have been obtained for the formulations using lightweight aggregates or bio-based materials in terms of regulating moisture levels, further studies are needed to assess the long-term stability of the performance obtained [86,87,91]. Therefore, performing accelerated aging tests and monitoring the performance in real-life conditions would allow for more accurate quantification of the evolution of the cooling potential over the lifespan of the paving stones.

From an operational point of view, the mechanical resistance results showed that most of the formulations were compatible with pedestrian and moderately dense public spaces. This is an essential aspect for the practical implementation of the paving stones, since it ensures that performance is not compromised in terms of functionality or safety. However, for other uses, such as vehicular traffic or logistics areas, optimization or compromise in terms of porosity/mechanical resistance may be considered.

The prospects for urban-scale applications are particularly favourable when considered as part of an integrated approach to the adaptation to climate change. Evaporative cooling pavers do not compete with other mitigation measures, such as vegetation or shading, but rather provide an alternative solution, especially when these measures are difficult to implement [92]. Additionally, the combination with alternative stormwater management systems offers noteworthy prospects for the use of rainwater to passively cool urban soils while at the same time mitigating runoff [87].

Finally, this study also points to the need to consider a multi-scale approach to evaluate the overall effect of self-cooling pavers. Although the tests performed allow for the precise quantification of the effects at the material and surface levels, future studies may aim to analyse the effects at the neighbourhood level, considering the interactions between surfaces, air, and urban activities. Such studies would allow for a more precise estimation of the potential gains to be obtained in terms of comfort, public health, and mitigation of the urban heat island effect. On the other hand, no economic assessment was carried out in the present study. Therefore, the financial feasibility of the proposed approach remains to be evaluated in future work.

To conclude, despite the limitations related to water management and sustainability, the evaporatively cooled pavers studied in this research have demonstrated significant potential for urban-scale applications to be integrated into urban planning development projects. Their capacity to enhance the urban microclimate, together with the relatively simple implementation and use of sustainable materials, renders this solution applicable and feasible to enhance the thermal resilience of urban areas to current and future climate change.

## 6. Conclusions

This study demonstrates that evaporative cooling efficiency in concrete pavers cannot be predicted solely from water absorption or total porosity, but depends on the functional coupling between capillarity, pore connectivity and thermal inertia. The experimental results obtained at the material scale and under real-world conditions allow us to move beyond a purely volumetric approach to porosity, often favored in the literature, in favor of an integrated thermo-hydraulic approach.

The tests showed that the complete substitution of natural sand with raw recycled sand leads to high capillarity (≈500 mg/cm^2^), but does not result in significant surface cooling, with maximum temperatures reaching 43–44 °C. This limited performance is attributed to poorly connected porosity, in which the water remains mostly trapped within the matrix, thus reducing effective evaporation. Conversely, formulations incorporating bio-based materials or lightweight aggregates exhibit a more open and hierarchical porosity, which promotes water migration to the surface and its gradual evaporation.

Paving stones incorporating hemp (fibers and shives) have shown a significant reduction in maximum temperatures, between 38 and 40 °C, associated with intermediate capillarity (≈360–410 mg/cm^2^) and a reduced density (≈1900 kg/m^3^). These formulations provide an effective compromise between water availability and limited heat storage, resulting in regulated hygrothermal behavior. Formulations based on expanded clay stand out for their even more stable thermal performance, with maximum temperatures of 37 to 39 °C and a marked reduction in day/night temperature fluctuations. The observed behavior may be consistent with differences in pore organization, but further pore-scale characterization would be required before attributing it to hierarchical porosity.

Conversely, formulations based on expanded glass, despite their low density, exhibit more modest thermal performance, confirming that their closed, low-absorption porosity does not allow for effective evaporative cooling. Comparison with reference materials highlights the significant gains achieved: while bitumen consistently exceeds 50 °C in summer conditions, some of the formulations developed allow for a reduction of 7 to 13 °C while maintaining mechanical strengths suitable for pedestrian traffic (20–30 MPa).

These results highlight three essential criteria for the design of self-cooling pavers: (i) sufficient but not excessive capillarity, (ii) interconnected porosity that promotes water migration to the surface, and (iii) moderate density ensuring a balance between thermal inertia and permeability. Formulations incorporating hemp or expanded clay simultaneously meet these requirements and appear to be the most relevant solutions among those studied. However, in the absence of in-depth characterization of the pore network, no direct conclusions can be drawn on the distribution, connectivity or hierarchy of pores in the formulations studied.

Beyond material performance, this study confirms the potential of evaporatively cooled pavers as a passive means of reducing overheating in urban soils, directly in contact with users. Their design using recycled and bio-based materials reinforces their environmental and social benefits, offering a solution compatible with climate adaptation strategies that are low in energy and resource consumption. By acting directly at ground level through passive evaporative mechanisms, the proposed paving materials may contribute to improving outdoor thermal comfort and reducing heat exposure in dense urban public spaces, particularly in contexts where access to active cooling solutions is limited.

The limitations of this work mainly concern the long-term management of moisture and the integration of foundation layers that provide water supply, which were intentionally excluded from this study. Future research should focus on the integration of paving stones with retention or drainage systems, as well as on the evolution of hygrothermal performance under traffic, aging, and climatic cycles. A neighborhood-scale evaluation, incorporating microclimatic simulations and extended in situ measurements, will ultimately allow for quantifying the actual impact of these paving stones on urban thermal comfort and the resilience of cities to heat waves.

## Figures and Tables

**Table 1:** Physical and descriptive properties of the raw materials used in this study.

Materials	Dimension [mm]	Bulk Density [kg/m^3^]	Water Absorption Capacity [wt.%]	Source/Origin
River sand	0–4	2700	1	Haute-Garonne department
Recycled sand	0–4	2180	9	Haute-Garonne department
Hemp fibers	20	50	360	Agrofibre—Local supplier
hemp shives	4–8	125	280	Agrofibre—Local supplier
Expanded clay	3–8	350	20	Laterlite—National supplier
Expanded glass	0.25–0.5	340	35	Poraver—European supplier

**Table 2:** Formulations tested (kg/m^3^ for each constituent).

Identification *	River Sand	Recycled Sand	Hemp Fibers	Hemp Shives	Expanded Clay	Expanded Glass	Cement	Metakaolin	Water
S0	1375		0	0	0	0	300	50	210
SR0	0	1375	0	0	0	0	300	50	210
SRC	0	1375	0	0	0	0	300	50	210
SRF0.1	0	1372	0.05	0	0	0	300	50	210
SRG1	0	1348	0	1.25	0	0	300	50	210
SRF0.1G1	0	1345	0.05	1.25	0	0	300	50	210
SRAG10	0	1237	0	0	0	17.3	300	50	210
SRAG20	0	1100	0	0	0	34.6	300	50	210
SRAC40	0	825	0	0	71.3	0	300	50	210
SRAC60	0	550	0	0	106.9	0	300	50	210

* S = river sand; SR = recycled sand; SRC = cribbed recycled sand; F = hemp fibers; G = hemp shives; AG = expanded glass; AC = expanded clay (Letters are derived from the French names). The associated number indicates the dosage by mass for the hemp and by volume for the expanded aggregates.

**Table 3:** Real condition test summary.

Formulation	Density (kg/m^3^)	Capillarity (mg/cm^2^)	T° max (°C)	Interpretation
Wood	-	-	35–37	Natural hygroscopic regulation
Bitumen	-	-	>50	Radiative absorption, no evaporation
SR0	1986	497	43–44	High absorption, blocked evaporation (trapped water)
SRF0.1	1938	361	39–40	Good moisture diffusion, net cooling effect
SRG1/SRG1F0.1	1895–1938	395–409	38–40	Gradual evaporation, hygroscopic regulation
AE40/AE60	1975–1883	302–187	37–39	Functional capillarity, optimal thermal inertia
P10/P20	≈1990	<200	40–42	Closed porosity, low evaporation

## Data Availability

The original contributions presented in this study are included in the article. Further inquiries can be directed to the corresponding author.
